# Synthesis of nanocrystals by discharges in liquid nitrogen from Si–Sn sintered electrode

**DOI:** 10.1038/srep17477

**Published:** 2015-12-01

**Authors:** H. Kabbara, C. Noël, J. Ghanbaja, K. Hussein, D. Mariotti, V. Švrček, T. Belmonte

**Affiliations:** 1 Université de Lorraine, Institut Jean Lamour, UMR CNRS 7198, NANCY, F-54042, France; 2 CNRS, Institut Jean Lamour, UMR CNRS 7198, NANCY, F-54042, France; 3 Faculty of Science, section III, Department of applied physics, Lebanese University, Tripoli, Lebanon; 4 Nanotechnology & Integrated Bio-Engineering Centre (NIBEC), University of Ulster, Shore Road, Newtownabbey, BT37 0QB, United Kingdom; 5 Research Center for Photovoltaic Technologies, National Institute of Advanced Industrial Science and Technology (AIST), Tsukuba, Ibaraki 305-8568, Japan

## Abstract

The synthesis feasibility of silicon–tin nanocrystals by discharges in liquid nitrogen is studied using a Si–10 at % Sn sintered electrode. Time-resolved optical emission spectroscopy shows that silicon and tin melt almost simultaneously. The presence of both vapours does not lead to the synthesis of alloyed nanocrystals but to the synthesis of separate nanocrystals of silicon and tin with average sizes of 10 nm. These nanocrystals are transformed into amorphous silicon oxide (am–SiO_2_) and β–SnO_2_ by air oxidation, after evaporation of the liquid nitrogen. The synthesis of an am-Si_0.95_Sn_0.05_ phase around large silicon crystals (~500 nm) decorated by β–Sn spheroids is achieved if the current flowing through electrodes is high enough. When the sintered electrode is hit by powerful discharges, some grains are heated and tin diffuses in the large silicon crystals. Next, these grains are shelled and fall into the dielectric liquid.

Si-Sn nanocrystals (NCs) have great potential for Li-ion batteries^1^ and photovoltaics cells^2^. The direct growth of Si–Sn alloys is often difficult because of the large difference in the lattice constants (~20%) of Si and α–Sn, and the low solubility of Sn in Si (~6–8 × 10^19^ cm^−3^, *i.e.* ~0.15 at % at 1100–1200 °C)^3^. At high Sn concentration, the equilibrium Si–Sn alloy is a two-phase mixture composed of the diamond-like Si phase and the Sn phase which transforms from the diamond-like α-Sn (grey tin) to tetragonal β-Sn (white tin) at 286.3 K (13.2 °C).

The optical band gap of Si–Sn NCs would be direct and not indirect like in silicon and it could be tuned since it depends on both the tin content and the NC size. According to Jensen *et al.*^4^, at 2.2% tensile strain, the band gap becomes direct with a magnitude of 0.85 eV. Increasing tin content up to 25 at % decreases almost linearly the bandgap^5^ whereas diminishing the NC size. For photovoltaics application as well as in optoelectronic^4^,^5^, such a material would find a wide utilization thanks to the possibility to precisely tune the energy levels and optoelectronic properties by the combined effects due to Si-alloying and quantum confinement.

Recently, the synthesis of Si–Sn NCs exhibiting quantum confinement properties was achieved by nanosecond laser ablation in water of an amorphous Si–Sn target^2^. Laser ablation in liquids (LAL) usually reaches yields of ~100 mg h^−1^. Resorting to electrical discharges in liquids might increase them up to 100 g h^−1^.

Submerged arcs generated in the liquid^6^,^7^,^8^,^9^,^10^,^11^ are characterized by spatial confinement with very high pressure, which might allow the growth of alloy NCs by chemical reaction. Practically, it turns out that if two electrodes with different materials are employed, NCs of each type of materials are produced but no alloy is formed^12^. Sintered targets are then preferred to get alloyed NCs^13^,^14^. By this technique, rather similar plasma conditions to ns laser ablation can be generated with nonetheless a significantly improved production yield. Another advantage lies in a better control and knowledge of plasma conditions, which can also help to better understand how these NCs are formed.

In this manuscript, we explore the possibility to synthesize Si–Sn NCs by discharges in liquid nitrogen which is an oxygen-free strong dielectric. In addition to characterization of NCs, time-resolved optical emission spectroscopy is used to investigate the dynamics of the plasma and to correlate it with material results. Special attentions are paid to target erosion and mechanisms which could lead to the synthesis of SiSn NCs.

## Results and Discussion

### Nanocrystals Characteristics

When the synthesized products are removed from liquid nitrogen, air oxidation contributes to convert metallic or semiconductor NCs into oxides (SnO_2_, SiO_2_ and (Si_1–x_Sn_x_)O_2_). Oxidation is relatively fast and we were unable to avoid it.

**Figure 1:**
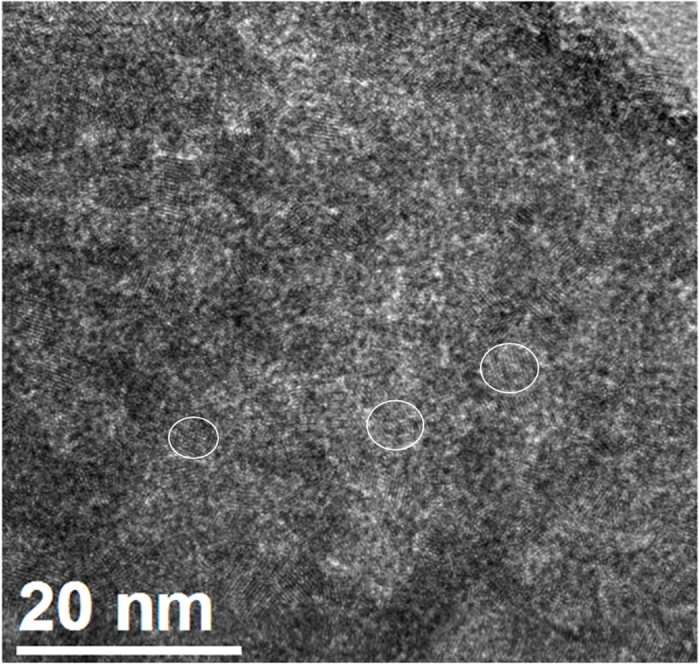
Large-view TEM image of a Si-Sn sample synthesized with a current of 1 A with examples (white circles) of crystalline domains.

**Figure 2:**
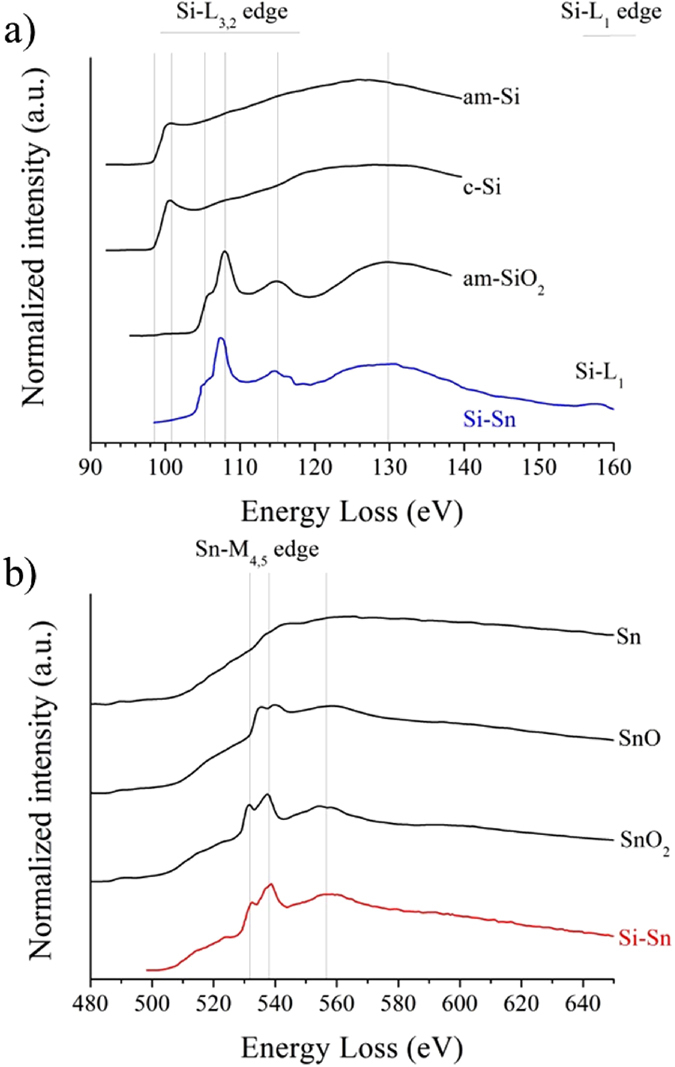
(**a**) EEL spectra at the Si-L_3,2_ edge of a representative sample synthesized with a current of 1 A. Reference spectra (amorphous and crystalline silicon and amorphous SiO_2_) taken from^4^ are given for comparison. (**b**) EEL spectrum at the Sn-M_4,5_ edge of a representative sample synthesized with a current of 1 A. Reference spectra taken from^5^ are given for comparison.

In Fig. 1, a large-view transmission electron microscope (TEM) image, which is representative of the analysed samples obtained with a current of 1 A, shows the presence of crystalline nanoparticles spread in an amorphous matrix. Identifying the various phases synthesized by pulsed discharges in liquid nitrogen is complex. Indeed, one might expect to find Si, α-Sn, β-Sn, SiO_2_, SnO_2_, Si_1–x_Sn_x_ and (Si_1–x_Sn_x_)O_2_. Unreported micro-energy dispersive spectroscopy (EDS) analysis shows that our samples contain in average 80.7 at %Si, 6.2 at %Sn and 13.1 at %O, giving a Sn/Si atomic ratio which is almost the same as in the target. To ease identification, electron energy loss spectroscopy (EELS) is used prior to indexation of micro-diffraction patterns. Figure 2a shows EEL spectra at the Si-L_3,2_ edge of a representative sample of NCs synthesized with a current of 1 A. The presence of amorphous SiO_2_ (am–SiO_2_) is observed in all samples, where various NCs are embedded. The EEL spectrum at the Sn-M_4,5_ edge indicates that tetragonal SnO_2_ (β–SnO_2_) NCs are present (Fig. 2b and see Supplemental Material 4 for high-resolution TEM images of diamond tetragonal β–SnO_2_ NCs), however no Si_1–x_Sn_x_ or (Si_1–x_Sn_x_)O_2_ can be observed. If silicon NCs were to be present, these would have been largely oxidized, making it difficult to detect the presence of any crystalline Si (c-Si) material. Indeed, air oxidation is efficient and turns Si NCs into amorphous^15^. Unreported results about discharges in liquid nitrogen between two crystalline silicon electrodes show clearly that Si NCs with larger diameters lying in the range 10–20 nm are synthesized. When diameters are large enough, oxidation is limited by the synthesis of a passive SiO_2_ outer shell, leaving an oxygen-free silicon core. These results agree well with those obtained by Kobayashi *et al.*^16^.

**Figure 3:**
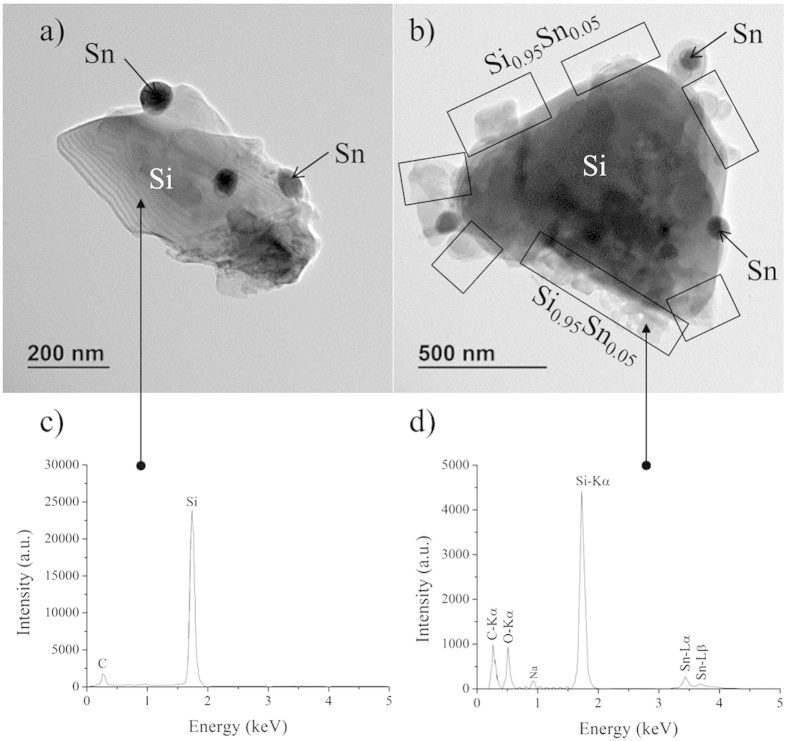
(**a**) TEM image of a Si grain decorated by Sn spheroids scratched from the sintered material. (**b**) TEM image of a Si grain decorated by Sn spheroids obtained after treatment. The Si_0.95_Sn_0.05_ layer is delimited by squares. (**c**) Micro-EDS spectrum corresponding to the silicon crystal in image (**a**). (**d**) Micro-EDS spectrum corresponding to the Si_0.95_Sn_0.05_ layer in image (**b**).

**Figure 4:**
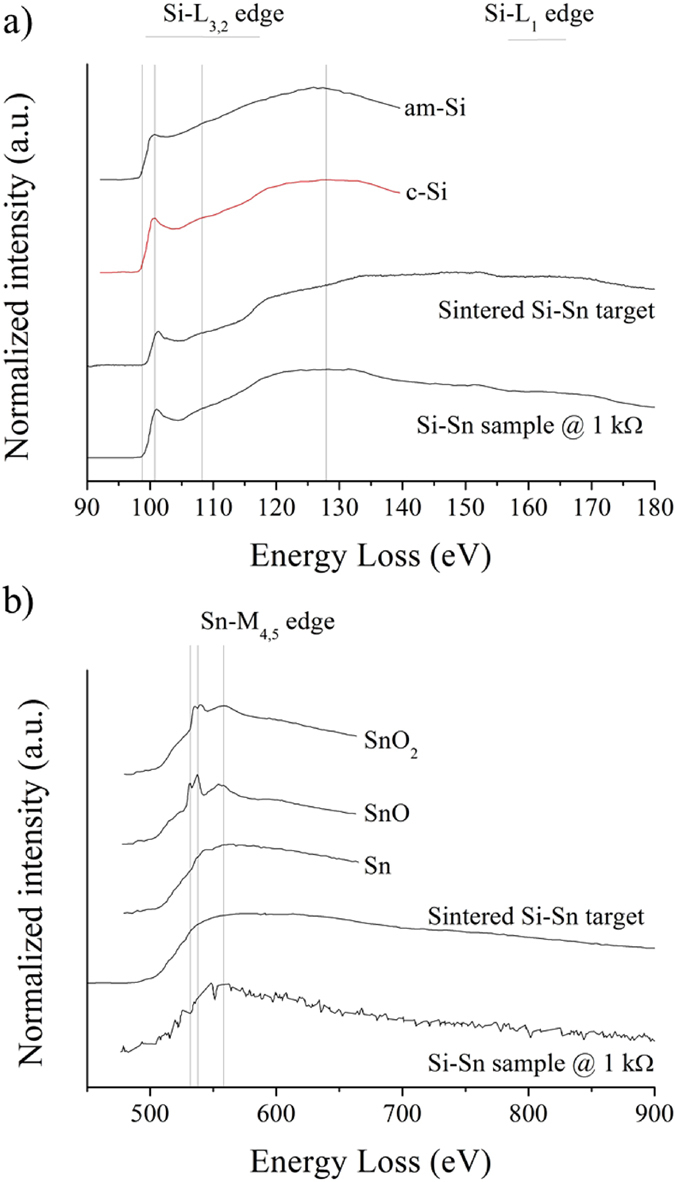
(**a**) EEL spectra at the Si-L_3,2_ edge of a representative sample synthesized with a current of 10 A. Reference spectra (amorphous and crystalline silicon and amorphous SiO_2_) taken from^4^ are given for comparison. (**b**) EEL spectrum at the Sn-M_4,5_ edge of a representative sample synthesized with a current of 10 A. Reference spectra taken from^5^ are given for comparison.

When the ballast resistance is only 1 kΩ, the current is 10 A and spark discharges are much stronger. The erosion mechanism of the sintered target is then completely different. Very large grains of silicon decorated by tin nanoparticles with diameters ranging from 50 to 70 nm are heated by the discharge and shelled. The mean size of the loose grains collected in these conditions corresponds to that of silicon grains of the target. In Fig. 3a,b, two grains are shown, one collected directly by scratching the target before treatment, and one collected by sedimentation after treatment. The grain in Fig. 3a shows only two phases: a large silicon crystalline grain decorated by tin nanoparticles. Micro-EDS analysis (Fig. 3c) shows that silicon does not contain any tin before treatment. The grain in Fig. 3b, obtained by sedimentation after treatment, shows three phases: a large silicon crystalline grain decorated by tin nanoparticles and an amorphous phase. The amorphous irregular layer contains up to 5 wt.% (~1.2 at %) tin after treatment (micro-EDS analysis in Fig. 3d). The amorphous nature is probably due to the very high tin content, which is about 8 times the solubility of tin in solid silicon at 1100–1200 °C^3^. The amorphous phase was likely formed by melting the outermost part of the silicon crystal followed by rapid quenching as seen in Fig. 3b (areas delimited by squares); this could not be observed in Fig. 3a. During the initial heating and melting, Sn from surrounding nanoparticles has alloyed with the outermost part of the large silicon grains yielding the amorphous alloyed Si-Sn layer. We infer that heating of the silicon grains by the spark discharge promotes the diffusion of tin into silicon, leading to the synthesis of a Si_1–x_Sn_x_ amorphous phase. While micro-EDS confirms the presence of Sn in the amorphous phase, no fingerprint of any Si_1–x_Sn_x_ phase is observed in EEL spectra which show only c-Si and Sn (Fig. 4). This is certainly due to the low amounts of Si_1–x_Sn_x_. It is interesting to note that irradiation of the amorphous Si-Sn layer by the TEM electron beam, induces tin segregation on the surface of the amorphous Si-Sn phase (see Supplemental Material 5), which further confirm the presence of alloyed tin. The TEM beam-induced segregation forms nano-sized (2–5 nm diameter) “*exsolved*” tin spheroids. The amorphous phase is expected to be far from thermodynamic equilibrium and the electron irradiation easily leads to the formation of two stable phases (silicon and tin), which could also be a reason for the absence of the Si-Sn EELS fingerprint.

In summary, depending on the ballast resistance and consequently on the discharge current, either nanocrystals of silicon and tin with size close to 10 nm are synthesized and further oxidized in air or grains are heated and shelled from the sintered target, leading to the synthesis of a small amount of a Si_1−x_Sn_x_ amorphous layer surrounding the large silicon crystals decorated by tin nanoparticles. To our knowledge, this latter mechanism is the first of its kind ever described. Applied to alloys with nano-sized grains, like those produced by mechanical alloying for instance, this method might give dispersed nanoparticles with proper size and composition. The discharge simply “scratches” the surface to shell grains and this explains why complex phase compositions can be kept.

### Synthesis characteristics

**Figure 5:**
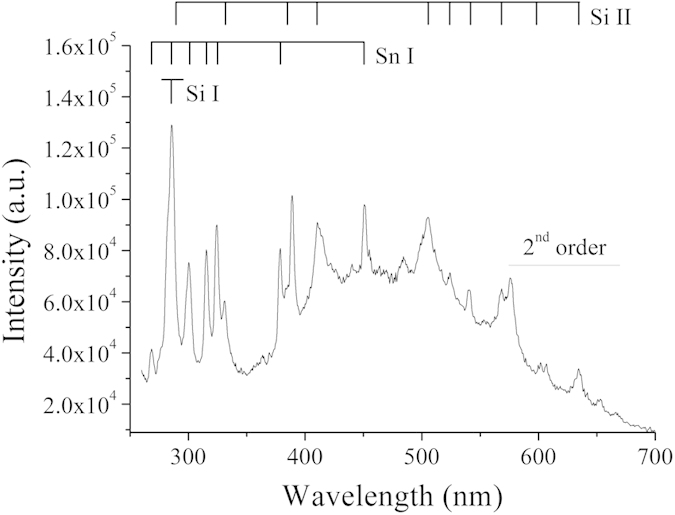
Visible range spectrum obtained with a Si-10 at %Sn target and a Si pin-electrode immersed in liquid nitrogen with a current of 1 A. Transition beyond 550 nm are second-order.

**Figure 6:**
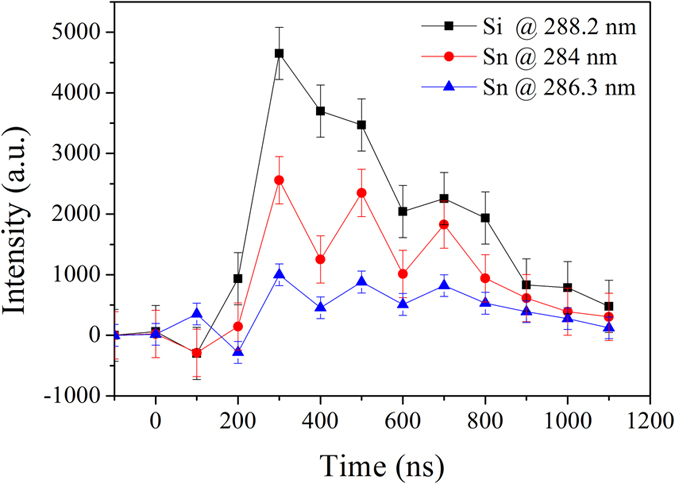
Time evolution of three selected lines observed between 282 and 292 nm.

Time-resolved optical emission spectroscopy was performed with a ballast resistance of 10 kΩ only (*i.e.* a current of 1 A). Si I, Si II and Sn I transition lines were observed (Fig. 5). Conversely, no N I lines were found. This lack of nitrogen lines was already reported^15^. Nitrogen seems not having a chemical role in the synthesis process. By selecting three lines appearing within a short range of wavelengths (see Supplemental Material 6)–one of Si I at 288.2 nm, and two of Sn I at 284 and 286.3 nm –, we could record the time evolution of these lines at one stroke (Fig. 6).

We notice that the silicon line appears slightly before or, say, at the same time as the emission of tin lines, although the corresponding transitions involved upper levels with almost identical energies (see Supplemental Material 6).

### Particles synthesis mechanism

This experimental result is rather unexpected. Indeed, the melting temperature of tin (505 K–232 °C) is much lower than that of silicon (1687 K–1414 °C). If the electrode surfaces were heated by radiation, a mechanism usually put forward to explain the formation of craters in conductive electrodes during electrical discharge machining^17^, the emission of the tin vapour should occur much sooner than the emission of the silicon vapour. Heating of silicon is then ensured by another mechanism.

At 77 K, the conductivity of intrinsic silicon is as low as 10^−42^ Ω^−1^ m^−1 ^18. Even doped, it is still about 10^−4^ Ω^−1^ m^−1 ^19 whereas the conductivity of tin is 5 × 10^3^ Ω^−1^ m^−1 ^20, still at 77 K. The ohmic current is negligible and only a strong displacement current flows through the silicon wafer acting as a capacitor. Then, heating is ensured by the Eddy currents, like in any induction system. It induces a fast temperature rise by local Joule effect, which is responsible for the early emission of silicon. Furthermore, the cooling of Sn by liquid nitrogen is more efficient than that of Si because of its higher thermal conductivity (60 W m^−1^ K^−1 ^21 vs 0.16 W m^−1^ K^−1 ^20 at 77 K). As no nanoparticles of Si_1–x_Sn_x_ are synthesized in these experimental conditions, the simultaneous presence of the two vapours does not lead to alloyed NCs, as expected. This confirms the difficulty to create from two different vapours alloyed nanoparticles, even if these vapours are emitted simultaneously.

**Figure 7:**
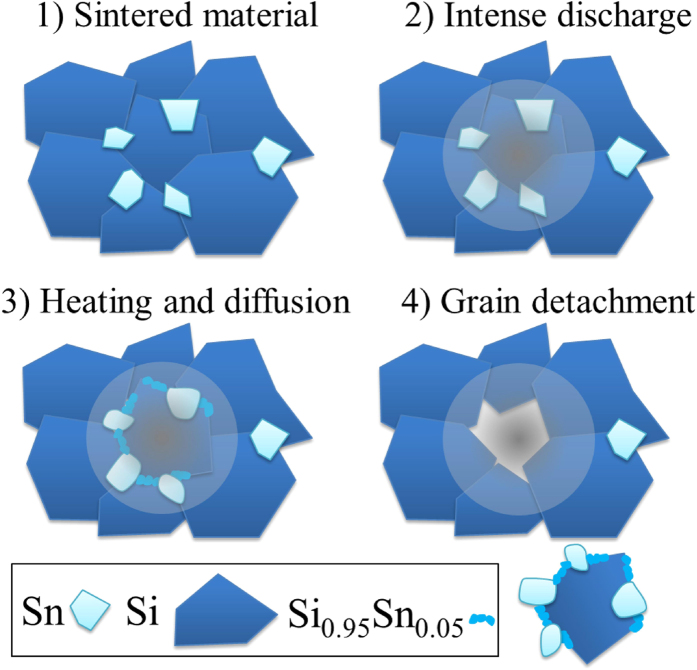
Proposed growth mechanism explaining how the Si_0.95_Sn_0.05_ phase at the edges of large silicon crystals (~500 nm) decorated by β–Sn spheroids are synthesized by high intensity discharges.

When the ballast resistance is only 1 kΩ, the current is 10 A and spark discharges are much stronger. The erosion mechanism of the sintered target is then completely different. Synthesis of Si_0.95_Sn_0.05_ in large silicon crystals (~500 nm) decorated by β–Sn spheroids is achieved as described in Fig. 7. To our knowledge, this latter mechanism is the first of its kind ever described. When the discharge hits the sintered material, very large grains of silicon decorated by tin spheroids are heated. Diffusion of tin in silicon is activated and the amorphous layer, made of Si_0.95_Sn_0.05_ around the silicon grain is synthesized. Because the discharge is very powerful, thermal gradients are likely strong enough to induce sufficient stress to shell the crystals. Moreover, the mean size of the loose particles collected in these conditions corresponds to that of silicon grains in the target, *i.e.* the sintered material. Then, it is important to stress here that alloying is achieved and permits the synthesis of a small amount of Si_1–x_Sn_x_ surrounding large silicon crystals and decorated by tin nanoparticles.

## Conclusion

Discharges in liquid nitrogen between a silicon pin-electrode and a sintered Si–Sn target gave unexpected results:

- Depending on the discharge current, either β-Sn nanocrystals embedded in a Si matrix are synthesized and oxidized in the air after evaporation of liquid nitrogen (high ballast resistance–low current) or large composite grains made of tin nanparticles and crystalline silicon are heated and shelled from the sintered target (low ballast resistance–high current),
- An amorphous phase containing up to 5 at %Sn is synthesised around the large silicon grains,
- Time-resolved optical emission spectroscopy shows Si I, Si II and Sn I transitions but no nitrogen lines. The absence of nitrogen in the nanoparticles is likely related to this specific behaviour.
- Silicon vapour is emitted slightly before tin vapour by a fast heating of silicon due to local joule effect.

No Si–Sn nanocrystals could be produced in the discharge, although vapours of both elements reach their maximum emission simultaneously. On the other hand, we showed the possibility of alloying Si and Sn in an amorphous phase with controlled composition by using the discharge as a tool to shell the grains of the sintered target. Our work shines light on important mechanisms taking place during pulsed discharges which are essential to tailor future synthesis processes. New experiments are now needed to study thoroughly the influence of the target microstructure and achieve highly controlled synthesis of alloyed NCs.

## Methods

The experimental setup used in this work is described in detail in one of our former works^15^ (see also Supplemental Material 1). Briefly, a DC high voltage power supply (Technix SR15-R-1200:15 kV–80 mA) fed a high-voltage solid-state switch (BEHLKE HTS-301-03-GSM) controlled by a low-frequency signal generator. A pulsed high voltage (PHV) of +10 kV was thus applied to the power electrode, the other electrode being grounded. The corresponding maximum current was either 10 A or 1 A according to the resistance of the ballast resistor: 1 kΩ or 10 kΩ, respectively. The on-time of one pulse was 200 ns. The operating frequency of the PHV was 3 Hz typically. No increase in the liquid temperature was measured even after 1000 successive discharges because the deposited energy in a single discharge is only ~10 μJ.

The setup was arranged in a pin-to-plate configuration. The inter-electrode distance was set at 100 ± 10 μm thanks to a micrometric screw. A silicon tip faced a Si-10 at %Sn cylinder (diameter: 10 mm–purity 99.999%) elaborated by R-DEC Co., Ltd. (Ibaraki, Japan) using spark plasma sintering. The curvature radius of the Si-tip, evaluated by optical microscopy, is about 100 μm. The pin-electrode was mounted on a XZ–stage to control its position relative to the plate-electrode. Thus, the inter-electrode gap distance could be set accurately and a free movement over a surface line was possible.

A typical x-ray diffraction (XRD) pattern (see Supplemental Material 2) shows that the sintered material was made of crystalline α-Sn, β-Sn and Si phases but no Si_1–x_Sn_x_ phase. The α-Sn phase was likely stabilized by epitaxial growth on silicon during the sintering process.

Optical emission spectroscopy was performed with a 550 mm focal length monochromator (Jobin–Yvon TRIAX 550) equipped with a 100 grooves mm^−1^ grating for overall spectra in the range 250–900 nm and a 1800 grooves mm^−1^ grating to record specific transitions at high spectral resolution. The spectrometer was coupled with a HORIBA Jobin–Yvon i-Spectrum Two iCCD detector. Each measurement is averaged over 25 spectra recorded 40 times (*i.e.* 1000 events) with an exposure time of 100 ns. After each of these 40 times, the pin-electrode had to be moved by about 100 μm to avoid the formation of a too large crater where the discharge could penetrate, which would limit the collected light. Even with this procedure, relatively noisy data were obtained. Recording the emitted light was only possible at low current (1 A). At high current (10 A), the erosion rate was too fast, leading to huge craters and weak observable emission (see Supplemental Material 3).

A double quartz-beaker forming a Dewar-like cell (volume: 80 cm^3^) was filled with standard liquid nitrogen (purity: 99.995%) provided by Air Liquide. On the bottom of the vessel, an aluminium substrate was deposited to collect the particles synthesized after 1000 successive discharges. These particles were transferred on a holey carbon film on a TEM copper grid simply by rubbing the surface of the aluminium substrate. Contamination by dissolved gases, dusts and other contaminants adsorbed on the vessel walls, as well as debris emitted by the surface or synthesized NPs, change the electrical conductivity of the liquid. The ageing of the liquid also affects the discharge itself. To limit this effect, the cell was cleaned and the liquid renewed between two series of discharges.

TEM investigation was performed on as-grown NCs with a JEOL ARM 200F–Cold FEG TEM/STEM running at 200 kV (point resolution 0.19 nm) fitted with a GIF Quatum ER. EELS experiments were performed in diffraction mode. The spectrometer is set to an energy dispersion 0.05 eV/channel. The condenser aperture, spectrometer entrance, camera length were respectively 150 μm, 2.5 mm and 4 cm leading to a collection half angle of 20 mrad and an energy resolution of 0.5 eV measured at full width at half maximum (FWHM) of zero loss peak.

## Additional Information

**How to cite this article**: Kabbara, H. *et al.* Synthesis of nanocrystals by discharges in liquid nitrogen from Si–Sn sintered electrode. *Sci. Rep.* **5**, 17477; doi: 10.1038/srep17477 (2015).

## Supplementary Material

Supplementary Materials
