# Supplementary material for: Synthesis of nanocrystals by discharges in liquid nitrogen from Si–Sn sintered electrode
Source: Sci Rep. 2015 Dec 1;5:17477. doi: 10.1038/srep17477 (PMC4664926; doi:10.1038/srep17477)
Supplement: Supplementary Materials [file srep17477-s1.docx]

**Synthesis of nanocrystals by discharges in liquid nitrogen**

**from Si**–**Sn sintered electrode**

**SUPPLEMENTAL MATERIAL 1**

H. Kabbara^1^, C. Noël^2^, J. Ghanbaja^1^, K. Hussein^3^, D. Mariotti^4^, V Švrček^5^ and T. Belmonte^2,*^

*^1^ Université de Lorraine, Institut Jean Lamour, UMR CNRS 7198, NANCY, F-54042, France*

*^2^ CNRS, Institut Jean Lamour, UMR CNRS 7198, NANCY, F-54042, France*

*^3^ Faculty of Science, section III, Department of applied physics, Lebanese University, Tripoli, Lebanon.*

*^4^Nanotechnology & Integrated Bio-Engineering Centre (NIBEC), University of Ulster, Shore Road, Newtownabbey, BT37 0QB, United Kingdom*

*^5^Research Center for Photovoltaic Technologies, National Institute of Advanced Industrial Science and Technology (AIST), Tsukuba, Ibaraki 305-8568, Japan*

*** corresponding author:** [thierry.belmonte@univ-lorraine.fr](mailto:thierry.belmonte@univ-lorraine.fr) (tel.: +33383584091)

**Fig. S1:** Experimental setup used to synthesize SiSn nanocrystals. A positive high voltage is applied to the pin electrode, the counter electrode being grounded. A DC power supply (Technix SR 300 W – 10 kV –80 mA) feeds a high voltage solid-state switch (HTS-310-03-GSM) that delivers a maximum current of 30 A for a voltage inferior to 10 kV. A ballast resistance of 1 or 10 kΩ is used to create either strong or soft discharges. The value of the ballast resistance determines the delivered electric charges, which sets the mean diameter of an impact (see [S1] for detail). The inter-electrode gap is set manually with a micrometric screw at 100 µm (± 10 µm). Electrodes are immersed in the liquid nitrogen. Electrical measurements were performed with two voltage probes. The first probe (Agilent N2771A 15 kV) has an attenuation factor of 1000, an input resistance of 1 GΩ, an input capacitance of 6 – 20 pF and a bandwidth of 50 MHz. The second probe (Agilent 10076A) has an attenuation factor of 100, an input resistance of 66.7 MΩ, an input capacitance of 3 pF and a bandwidth of 250 MHz. This probe is used to measure the voltage drop across a resistance (12.5 Ω) to obtain the time evolution of the current flowing in the plasma. These electrical characteristics are visualized and saved on an Agilent Technologies, infiniium 54832B DSO oscilloscope (maximum acquisition frequency: 4 GSa/s – bandwidth: 1 GHz).

[S1] A. Hamdan, C. Noel, F. Kosior, G. Henrion and T. Belmonte, Impacts created on various materials by micro-discharges in heptane: Influence of the dissipated charge, J. Appl. Phys. 113 (2013) 043301

**SUPPLEMENTAL MATERIAL 2**


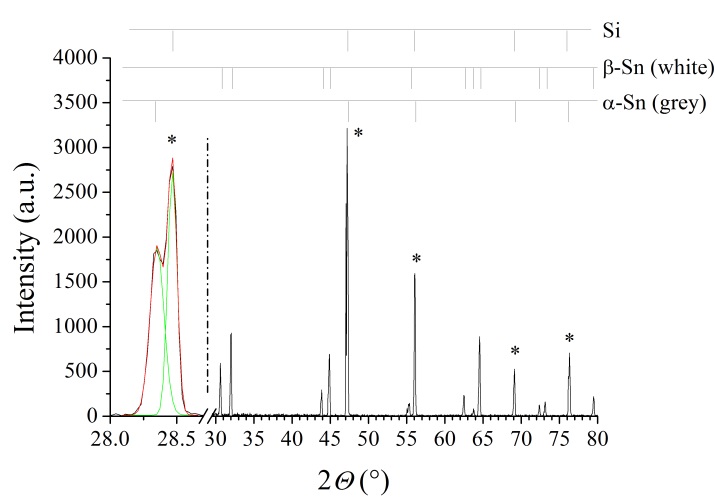


**Fig. S2:** XRD pattern of the sintered SiSn target. Peaks with star are double. The X–scale is expended around 28.3° to show such a double peak. We clearly observe the presence of α and β–tin. The presence of α–tin is assumed to be due by an epitaxial growth on silicon during the sintering process.

**SUPPLEMENTAL MATERIAL 3**


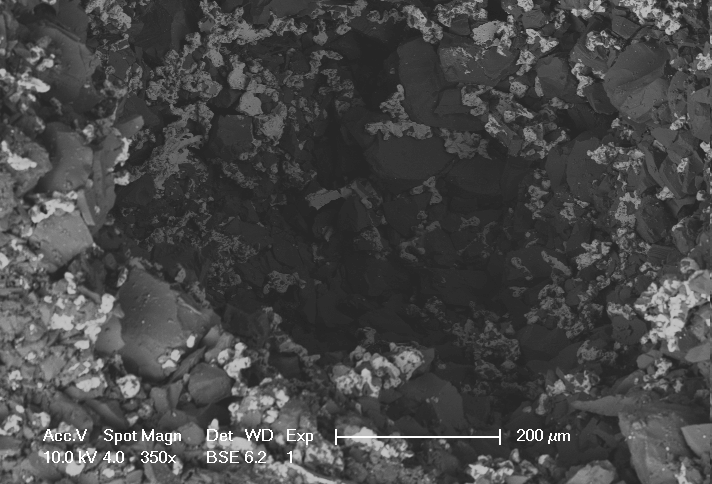


**Fig. S3:** SEM image in back-scattered electron mode of a Si–10.at%Sn sintered target showing a large hole (~500 µm in diameter) created by 1000 discharges with a 10 A current. Light areas are due to tin, dark ones to silicon. The successive discharges drill huge craters. The emitted light by a discharge is such a crater is trapped and cannot be collected easily with an optical fibre to perform optical emission spectroscopy

**SUPPLEMENTAL MATERIAL 4**


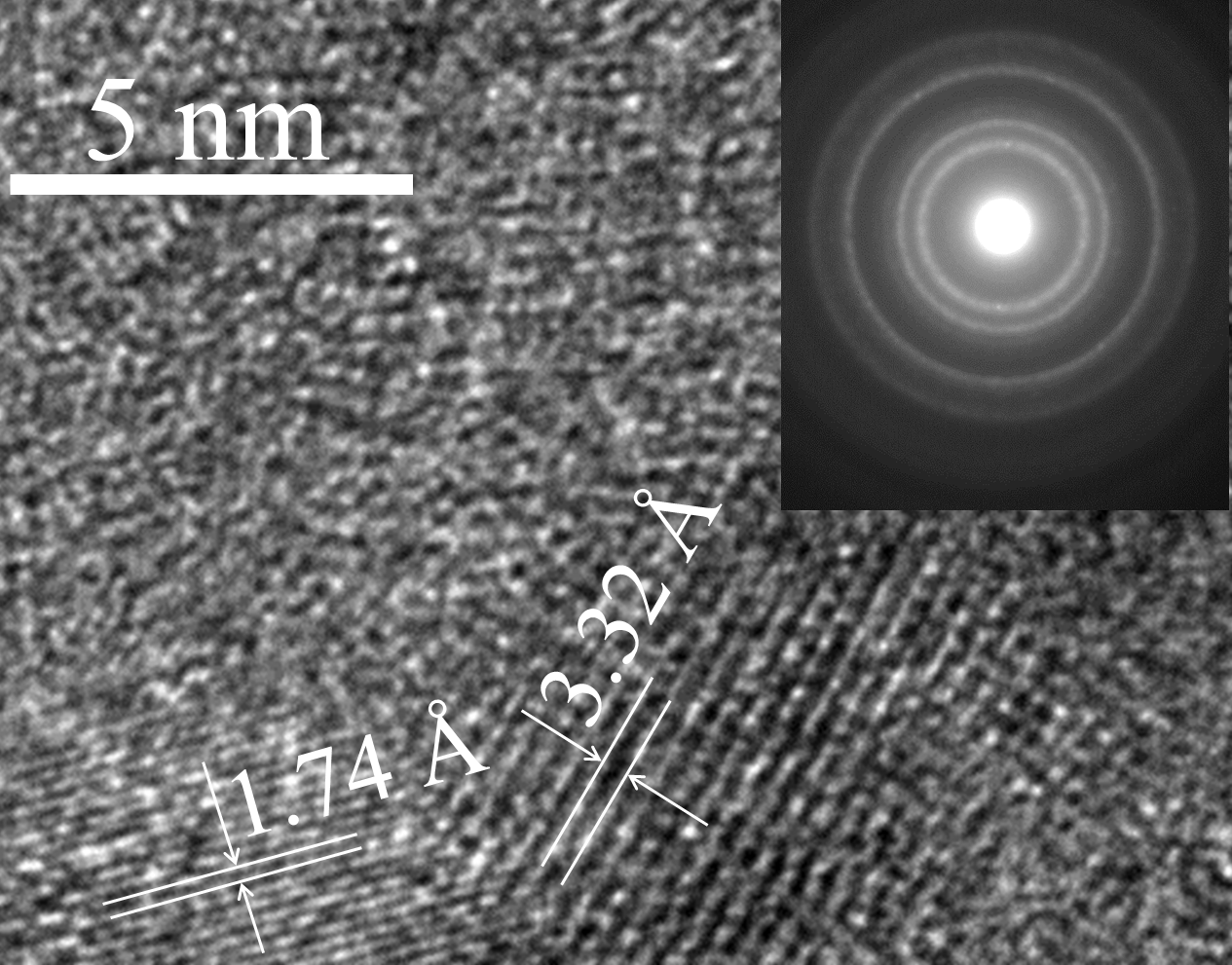


**Fig. S4.** High-resolution TEM image of two β-SnO_2_ NCs. The interplanar spacings are 1.74 Å (theoretical value is 1.74 for (211)) and 3.32 Å (theoretical value is 3.35 for (110)) –these values are averages determined over at least 10 rows–. Selected area electron diffraction pattern showing the (110), (101), (111), (211) and (112) planes of β-SnO_2_.

**SUPPLEMENTAL MATERIAL 5**

**
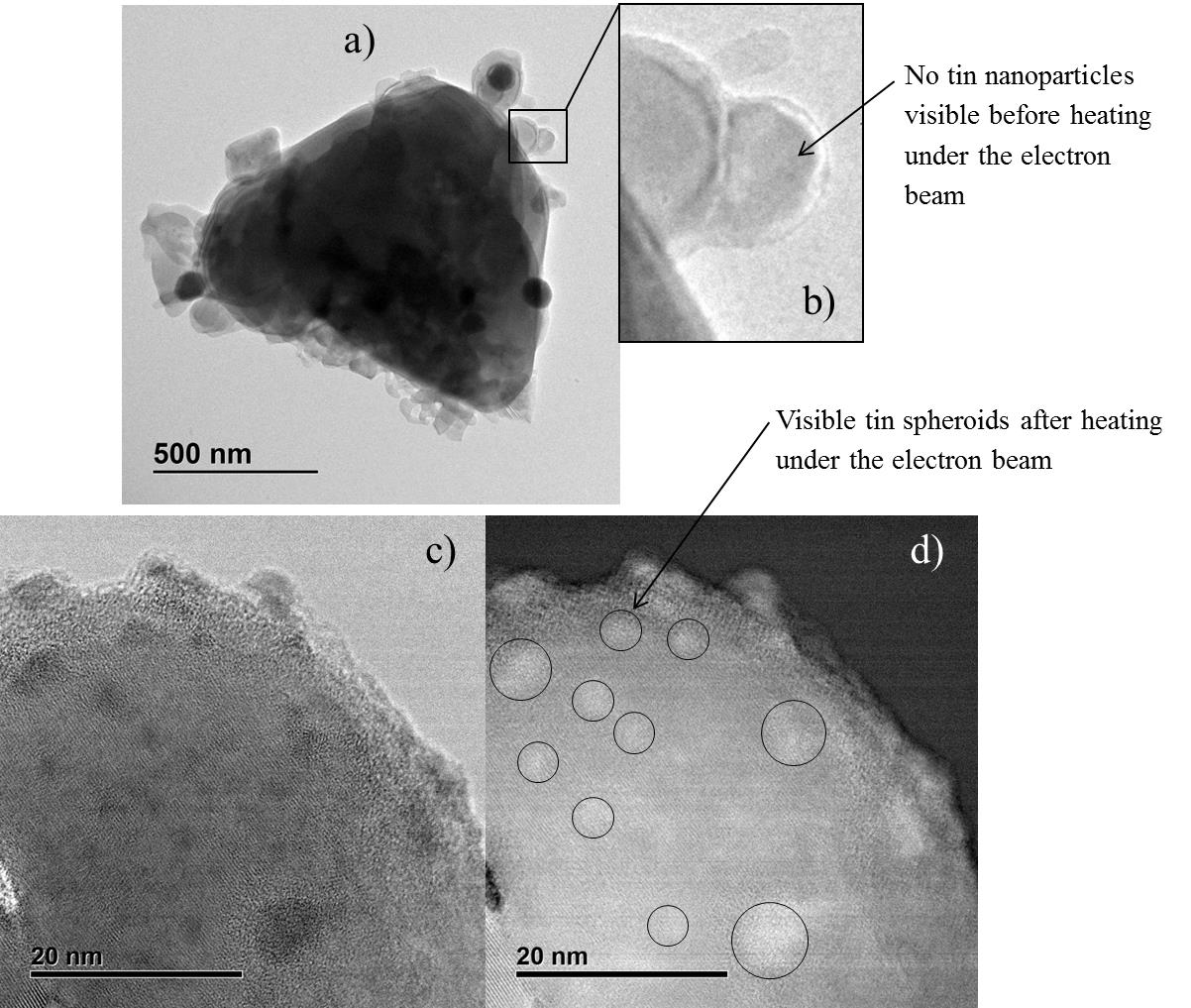
**

**Fig. S5.** a) TEM image of a Si grain decorated by Sn spheroids obtained after treatment. b) magnification of the edge (the Si_0.95_Sn_0.05_ phase) irradiated by the electron beam. c) High-resolution image of the irradiated area where tin is segregated. d) Corresponding STEM image.

**SUPPLEMENTAL MATERIAL 6**

**
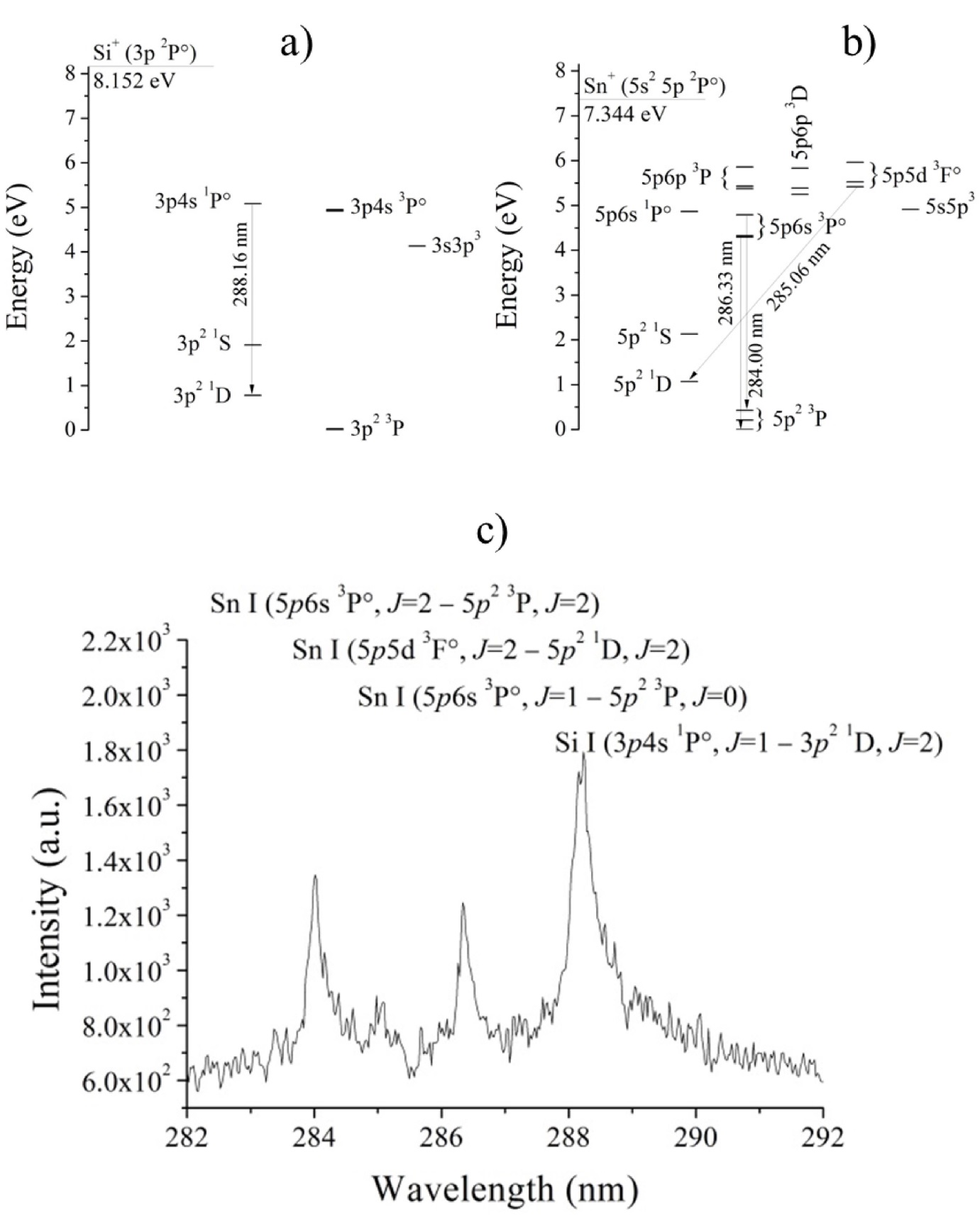
**

**Fig. S6:** a) Energy diagram of Si. b) Energy diagram of Sn. Both diagrams are very similar, silicon and tin having the same electronic structure. c) Selected transition lines observed between 282 and 292 nm – they are depicted in a) and b).
